# The Association of Environmental Tobacco Smoke Exposure and Inflammatory Markers in Hospitalized Children

**DOI:** 10.3390/ijerph16234625

**Published:** 2019-11-21

**Authors:** E. Melinda Mahabee-Gittens, Ashley L. Merianos, Patricia C. Fulkerson, Lara Stone, Georg E. Matt

**Affiliations:** 1 Division of Emergency Medicine, Cincinnati Children’s Hospital Medical Center; University of Cincinnati College of Medicine, Cincinnati, OH 45229, USA; lara.stone@cchmc.org; 2 School of Human Services, University of Cincinnati, Cincinnati, OH 45221, USA; ashley.merianos@uc.edu; 3 Division of Allergy and Immunology, Cincinnati Children’s Hospital Medical Center, University of Cincinnati; College of Medicine, Cincinnati, OH 45229, USA; pcfulkerson@gmail.com; 4 Department of Psychology, San Diego State University, San Diego, CA 92123, USA; gmatt@sdsu.edu

**Keywords:** cotinine, inflammatory markers, cytokines, secondhand smoke exposure, children

## Abstract

Background: Environmental tobacco smoke (ETS) exposure is associated with altered cytokine levels in children. We sought to examine ETS exposure prevalence and the relationship between ETS exposure and cytokine levels in a sample of hospitalized children. (2) Methods: Inflammatory markers (IL-8, IL-1β, IL-10, and TNF-α) and cotinine were measured in saliva of hospitalized, nonsmoking children (N = 112). To assess the association between ETS exposure and immune system response, we built a multivariate regression model including the four inflammatory markers as the response variables and cotinine, age, sex, and discharge diagnosis as explanatory variables while assessing possible interaction effects. (3) Results: Mean age (SD) was 5.8(5.0) years; Geometric Mean (GeoM) cotinine = 1.8 [95% CI = 1.4–2.2]. Children with non-inflammatory other diagnoses had lower IL-10 (*p* = 0.003) and TNF-α (*p* = 0.009) levels than children with inflammatory other diagnoses. Children with asthma (*p* = 0.01) and bacterial illnesses and/or pneumonia (*p* = 0.002) had higher IL-8 levels. Independent of diagnosis, there was a significant curvilinear association between cotinine and IL-1β (*p* = 0.002) reflecting no association for cotinine levels <5 ng/mL and a positive association for >5 ng/mL. (4) Conclusions: Children with higher ETS exposure levels have higher IL-1β levels regardless of age, sex, and diagnosis. ETS exposure may increase pro-inflammatory immune responses in children and may interfere with native immune responses and the ability to heal and fight infection.

## 1. Introduction

Children who are involuntarily exposed to environmental tobacco smoke (ETS) are at increased risk for respiratory and infectious illnesses such as asthma, bronchiolitis, and otitis media [1,2]. The prevalence of ETS exposure remains high at 38% for young children; rates are even higher in some racial/ethnic and other subgroups [3]. Exposure to ETS and nicotine in children affects cell-mediated and humoral immune responses by augmenting the production of some pro-inflammatory cytokines and by decreasing the production of anti-inflammatory cytokines [4,5,6]. Prior studies have yielded conflicting results on which specific cytokines are affected by ETS exposure in children. For example, studies have demonstrated that ETS exposure results in elevated levels of pro-inflammatory cytokines (e.g., IL-8, IL-1β, TNF-α, IL-1) that may be associated with severe airway inflammation often seen in asthma. ETS exposure may also suppress cell-mediated responses with the production of anti-inflammatory cytokines (e.g., IL-10, IFN-γ), which may be protective against asthma and viral and bacterial pathogens [5,6,7,8,9]. A better understanding of these immune responses may explain why some ETS-exposed children have an increased incidence and severity of respiratory and infectious illnesses, including asthma, respiratory syncytial virus, and pneumonia and why others do not.

Prior research has examined levels of inflammatory markers measured in the blood and saliva samples of healthy children or children with minor illnesses who were exposed to ETS [8,9,10]. We sought to add to this area of research by examining cytokine levels in saliva samples of ill, hospitalized, nonsmoking children with differing diagnoses and ETS exposure levels. Our objectives in this pilot study were to examine: 1) the prevalence of ETS exposure and associated sociodemographic characteristics within a sample of hospitalized children; 2) the use of saliva as a biological source in which to examine cytokine levels in ill children and the relationship between ETS exposure and levels of specific cytokines; and 3) the relationship between clinical diagnoses and levels of specific cytokines.

## 2. Materials and Methods

### 2.1. Subjects

A convenience sample of 0–17-year-old patients (N = 199) who were hospitalized on general pediatric medical units between December 2016–May 2017 and who had documentation of positive or negative ETS exposure status in their electronic health record (EHR) were recruited. Previous research with this population of children demonstrates a high prevalence of ETS exposure [11,12]. Children were excluded if they had a tracheostomy (*n* = 2) or were smokers (*n* = 10). Caregiver consent and child assent on children age >11 were obtained. 

### 2.2. Measures

Caregivers reported sociodemographics (i.e., age, sex, race/ethnicity, insurance status, household income level, caregiver education level), and children’s EHRs were abstracted for ICD-10 discharge diagnosis. The discharge diagnoses were evaluated, and similar and clinically relevant diagnoses were grouped into five categories for analysis (by Dr. Mahabee-Gittens with verification by Dr. Fulkerson): 1) asthma or asthma/wheezing (children who had a discharge diagnosis of wheezing were included in this category if they were >2 years old); 2) viral or bronchiolitis/wheezing (children who had a discharge diagnosis of wheezing were included in this category if they were <2 years old); 3) bacterial illness and/or pneumonia; 4) non-inflammatory other illnesses (e.g., failure to thrive, constipation, psychiatric illness), and 5) inflammatory other illnesses (e.g., Kawasaki’s disease, allergic reaction, urticaria, viral pneumonia). The study was approved by our hospital’s Institutional Review Board.

### 2.3. Biological Assays

Saliva samples were obtained on all nonsmoking children and tested for cotinine, a major metabolite of nicotine [13], using enzyme-linked immunosorbent assay (ELISA) techniques by Salimetrics LLC [14]. Saliva was obtained, on average, 18.72 hours (SD: 0.68 hours) after admission. Unstimulated saliva samples were obtained by having the child suck or chew on a cotton sorbette [14] for a minimum of one minute until it was sufficiently moist. Sorbettes were then immediately placed into a test tube, transported on ice to the lab, centrifuged for 15 minutes, and then nondiluted saliva samples were frozen at −80 degrees Celsius until analyses were conducted. Two test results were obtained for each participant and we combined and averaged these two test values for analyses. These two cotinine values had high internal consistency with an intra-class correlation coefficient (ICC) of 0.996. The limit of quantitation (LOQ) was 0.15 ng/mL and for each value that was below the LOQ (*n* = 3), we used one-half the LOQ (0.075 ng/mL). Children with cotinine values >1.0 ng/mL were classified as having positive biochemical verification of ETS exposure [15,16,17]. 

Saliva samples were also tested using a Luminex Multiplex Assay for the following panel of inflammatory markers which were chosen based on prior similar studies [8,9]: IL-1β, IL-10, IL-8, TNF-α, IFN-γ, IL-4, and CRP. Similar to cotinine, two test values for each inflammatory marker were obtained for each participant, and we combined and averaged these two test values for analyses. We were able to reliably measure levels of IL-1β (ICC = 0.957), IL-10 (ICC = 0.926), IL-8 (ICC = 0.993), and TNF-α (ICC = 0.991) in the saliva of children in this study. The LOQs were: 0.80 pg/mL for IL-1β, 1.60 pg/mL for IL-10, 1.80 pg/mL for IL-8, and 1.20 pg/mL for TNF-α. However, we were unable to analyze the results of IFN-γ and CRP because of a large proportion of both test values <LOQ (*n* = 23, 20.5% and *n* = 93, 83.0%, respectively). We were unable to analyze the results of IL-4 due to the unreliable nature of the data resulting from large discrepancies between the two replicate measures.

Of the 199 participants, we had sufficient volumes of saliva for N = 113 (56.8%) to measure IL-1β, IL-10, IL-8, and TNF-α. One of these children with cytokine values did not have a cotinine value, and therefore, 112 (56.3%) were included in the analysis. We used Chi-square tests and independent sample t-tests to compare sociodemographic differences between participants who did and did not have enough saliva to have cotinine assessed. There were differences between hospitalized children who were included in the analysis because they had sufficient saliva volumes to obtain cytokine and cotinine values (*n* = 112) compared to those who did not have sufficient saliva (*n* = 87) based on child age (*p* = 0.001), sex (*p* = 0.01), and insurance type (*p* = 0.04). Children 0–1 years old (36.6%), 5–9 years old (25.0%), and 10–17 years old (29.5%) had higher rates of being included in the present analysis than children 2–4 years old (8.9%). Additionally, males (58.9%) and those with public health insurance (58.0%) had higher rates of having sufficient saliva volumes for analysis than females (41.1%) and those with private health insurance (42.0%). No differences were found based on child race, household income level, and discharge diagnosis.

### 2.4. Statistical Analyses

Salivary cotinine and the four inflammatory marker distributions were assessed for normality and underwent logarithmic transformation prior to all analyses to stabilize residual variances and control for positively skewed distributions. We present geometric means (GeoM), 95% confidence intervals (CIs), medians, and interquartile ranges (IQRs). We examined the relationship between patient characteristics and log-transformed cotinine by building a series of univariate linear regression models. We assessed bivariate correlations between child age, log-cotinine, and log-cytokine values via Pearson correlations. We performed similar analyses as Matsunaga et al. [9], using log-cotinine values to determine percentile cut points to assess if there was a dose-response threshold in the associations with TSE and cytokine levels. Specifically, we conducted independent t-tests to assess differences in cytokine values between the lower 25th percentile (0.05–0.59 ng/mL) vs. the upper 75th percentile of log-cotinine values (2.9–29.2 ng/mL). To explore potential nonlinear associations between cotinine and cytokine levels, we examined linear and quadratic polynomial regression terms and then examined the robustness of nonlinear associations plotting data and evaluating the model fit, using Cook’s Distance to identify influential data points, and re-estimating models excluding potentially influential data points. To assess the association between the four inflammatory markers and cotinine (all log-transformed), we built a multivariate regression model including log-IL-8, log-IL-1β, log-TNF-α, and log-IL-10 as the response variables and log-cotinine, child age, sex, and discharge diagnosis as explanatory variables while assessing possible interaction effects since these may influence inflammatory markers. The Type I error was set at α = 0.05 (nondirectional). All analyses were conducted using R (version 3.6.0) and SPSS (version 24.0).

## 3. Results

### 3.1. Demographics and Diagnoses

The mean age (SD) was 5.8 (5.0) years ranging from 0.04–17 years (median = 5.0, IQR = 0.7–10.0); 41.1% (*n* = 46) were females; 77.7% (*n* = 87) White, 15.2% (*n* = 17) African-American, and 7.1% (*n* = 8) other ethnic/racial backgrounds (Table 1). Over one-third (34.8%; *n* = 39) had a viral or bronchiolitis/wheezing discharge diagnosis followed by 23.2% (*n* = 26) with a bacterial illness and/or pneumonia diagnosis, 21.4% (*n* = 24) with an asthma or asthma/wheezing diagnosis, 17.0% (*n* = 19) with a non-inflammatory other diagnosis, and 3.6% (*n* = 4) with an inflammatory other diagnosis.

### 3.2. Prevalence of ETS Exposure and Associated Characteristics

Sixty (53.6%) children had positive biochemical verification of ETS exposure (i.e., >1.0 ng/mL). The range of cotinine levels was <0.15 ng/mL to 29.2ng/mL; GeoM = 1.8 [95%CI = 1.4–2.2]; median = 1.1, IQR = 0.6,2.9. There was a negative correlation between log-cotinine and child age (r = −0.273, *p* = 0.004). When cotinine levels were examined by child age group in an unadjusted linear regression model, we found that children who were 0–1 years old (GeoM = 2.6) had significantly higher mean cotinine levels than children who were 5–9 years old (GeoM = 1.4, *p* = 0.03) and 10–17 years old (GeoM = 1.2, *p* = 0.006; see Table 1). Children with public insurance or who were self-pay (GeoM = 2.7) had significantly higher mean cotinine values compared to children with commercial insurance (GeoM = 0.8, *p* < 0.001). Similarly, children with a household income level <$15,000 had significantly higher mean cotinine values (GeoM = 3.5) than those with a household income level >$15,000 (GeoM = 1.3, *p* < 0.001).

### 3.3. Relationship between Clinical Diagnoses and Cytokines

We found that hospitalized, nonsmoking children with non-inflammatory other diagnoses had significantly lower IL-10 (*p* = 0.003) and TNF-α values (*p* = 0.009) compared to children with inflammatory other diagnoses (Table 2). IL-8 results showed that hospitalized children with an asthma (*p* = 0.01) or bacterial/pneumonia diagnosis (*p* = 0.002) had significantly higher IL-8 values than those with inflammatory other diagnoses.

### 3.4. The Use of Saliva as a Biological Source to Measure Cytokines and the Relationship between ETS Exposure and Cytokines

In decreasing order, GeoM [95% CI] of the pro-inflammatory cytokines (pg/mL) were: IL-8: 611.0 [503.3−741.8]; IL-1β: 177.9 [143.0−221.1]; TNF-α: 7.1 [5.9−8.5], and GeoM [95% CI] of the anti-inflammatory cytokine (pg/mL) IL-10 was 1.2 [1.0–1.5]. There was an overall positive bivariate linear correlation between log-cotinine and log-IL-1β (r = 0.225, *p* = 0.02); see Table 3. There were no correlations between log-cotinine and log-transformed levels of IL-10, IL-8, or TNF-α.

Concerning our analyses to assess whether there was a dose-response threshold in associations with TSE and cytokine levels, we found no differences in IL-1β, IL-10, IL-8, TNF-α values between the lower 25th percentile of log-cotinine values (*n* = 28; 0.05−0.59 ng/mL) versus the upper 75th percentile of log-cotinine values (*n* = 28; 2.9−29.2 ng/mL; Table 4). To further investigate this relationship, we performed a sensitivity analysis and used Matsunaga and colleagues’ [9] cut point values based on their lowest (*n* = 9; 0.1−0.3 ng/mL) and highest (*n* = 23; 2.1−6.9 ng/mL) quartiles, which were lower than our values. Of note, the cotinine and cytokine levels were tested in serum in Matsunaga et al.’s work. We also report no differences between the log-transformed cotinine concentrations and inflammatory markers.

Because individual cytokine levels are the result of complex systemic immune responses, we considered nonlinear associations with cotinine to reflect a possible threshold of ETS exposure before an immune response is triggered, an asymptote or maximum immune response above a certain level of ETS exposure, or a change in direction of the association as ETS exposure increases from a lower to a higher level. We found a nonlinear relationship between cotinine and IL-1β. Further investigation revealed that there was no association between cotinine and IL-1β for cotinine levels less than approximately 5 ng/mL, but a positive association was found for cotinine values larger than approximately 5 ng/mL.

The association between ETS exposure and the four cytokines was examined in a multivariate regression model that allows for linear and quadratic associations of ETS exposure. We included child age, sex, and discharge diagnosis as additional explanatory variables and considered interactions of these variables with ETS exposure. Because child age and sex did not moderate the relationship between ETS exposure and any of the four cytokine variables and did not show any main effects, these two variables were removed from the model. The final model included cotinine (linear and quadratic terms) and IL-1β (R^2^ = 0.24, F = 5.41, *p* < 0.001), IL-10 (R^2^ = 0.12, F = 2.32, *p* = 0.04), IL-8 (R^2^ = 0.15, F = 3.05, *p* = 0.009), and TNF-α (R^2^ = 0.19, F = 4.12, *p* = 0.001). The model showed that ETS exposure, assessed by cotinine levels, was a significant predictor of IL-1β independent of diagnoses (Table 3) but showed no association with IL-8, IL-10, and TNF-α.

### 3.5. Robustness of Multivariate Regression Model Findings

To examine the robustness of our model estimates in general and the quadratic association in particular, we determined Cook’s D and identified cases with unusual influence. We re-estimated the IL-1β model while individually removing each of the five cases with high Cook’s D values (ranging from 0.11–0.30). The statistical significance of the association between log-cotinine and log-IL-1β only changed in one model (R^2^ = 0.20, F = 4.37, *p* = 0.001) that removed one of the five individual cases where the linear log-cotinine linear term was no longer significant ($\hat{\beta }$ = −0.87, *p* = 0.07 and quadratic term $\hat{\beta }$ = 0.86, *p* = 0.02). However, when removing all five cases simultaneously from the model, there were no differences in magnitude or statistical significance between log-cotinine and log-IL-1β (R^2^ = 0.18, F = 3.69, *p* = 0.002; cotinine linear term $\hat{\beta }$ = −1.44, *p* = 0.007 and quadratic term $\hat{\beta }$ = 1.43, *p* = 0.002) compared to the original model including all cases.

Regarding the model assessing the relationship between log-cotinine and log-IL-10, there were no changes in the magnitude or statistical significance while removing each of the two cases with high Cook’s D values (0.22 and 0.45) separately from the model. However, when excluding both cases simultaneously, the re-estimated model showed that log-cotinine (linear and quadratic terms) was a significant predictor of log-IL-10 independent of diagnoses (R^2^ = 0.09, F = 1.64, *p* = 0.15; cotinine linear term $\hat{\beta }$ = −0.63, *p* = 0.03 and quadratic term $\hat{\beta }$ = 0.52, *p* = 0.02). Therefore, we found additional statistical significance between log-cotinine and log-IL-10 independent of diagnoses when excluding these two influential data points.

The magnitude or statistical significance did not change while removing the four high Cook’s D values (ranging from 0.07–0.10) individually from the log-IL-8 model. While removing all four cases simultaneously in the log-IL-8 model (R^2^ = 0.13, F = 2.44, *p* = 0.03; cotinine linear term $\hat{\beta }$ = −0.78, *p* = 0.08 and quadratic term $\hat{\beta }$ = 0.69, *p* = 0.04), the quadratic term of log-cotinine became significant. 

We also re-estimated the TNF-α model, and while removing the three high Cook’s D values (ranging from 0.10−0.25) individually and then removing them altogether, there were no changes in the magnitude or statistical significance (R^2^ = 0.17, F = 3.39, *p* = 0.004; cotinine linear term $\hat{\beta }$ = −0.52, *p* = 0.20 and quadratic term $\hat{\beta }$ = 0.54, *p* = 0.10) compared to the original multivariate regression model results.

## 4. Discussion

To our knowledge, this is the first study to evaluate the salivary cytokine levels in ill, hospitalized, nonsmoking children who were highly exposed to ETS. Compared to other research examining cytokine levels and ETS exposure in children, levels of ETS exposure in our study were high; 56.3% of children were classified as exposed to ETS, which is a higher rate than that of the general population [3]. Of note, cotinine levels of >10 ng/mL are found in the saliva of heavy passive smokers and infrequent regular smokers [18], and 9% (*n* = 10) of children exposed to ETS in this study were at or over the level of 10 ng/mL. We demonstrated that inflammatory markers are measurable in the saliva of hospitalized children with concentrations in decreasing order: IL-8, IL-1β, TNF-α, and IL-10. These findings are consistent with a study that included healthy children and children with minor illnesses which demonstrated that IL-1β and IL-8 were measurable in saliva and present at much higher concentrations than the other pro-inflammatory markers assessed, IL-6 and TNF-α [10]. Thus, our results support the use of saliva as a minimally-invasive source in which to assess cytokine levels in hospitalized children.

We found a positive bivariate association between cotinine and the pro-inflammatory cytokine IL-1β among hospitalized children who were exposed to ETS. We did not observe an immunosuppressive effect with ETS exposure and IL-1β, but we observed higher IL-1β levels in children with higher ETS exposure. Since IL-1β is a marker of innate immunity activation [4], the presence of higher levels in children with higher ETS exposure indicates that there was likely elevated baseline inflammation possibly due to ETS exposure or that high levels of ETS exposure promoted an exaggerated inflammatory response to their illness with elevated IL-1β release. Laboratory studies have shown that exposure to cigarette smoke increases the production of IL-1β [4,19]. Since IL-1β induces the activation of macrophages and the release of neutrophils, the release may be involved in sustaining airway inflammation [4]. Further, prior studies indicate that there is marked up-regulation of IL-1β and IL-6 in hospitalized children with serious viral infections, such as H1N1 influenza, suggesting that IL-1β may be responsible for molecular reactions that lead to airway inflammation and increased disease severity [20]. We did not observe increased IL-1β levels with lower levels of ETS exposure, which may be because a threshold of ETS exposure is needed before up-regulation occurs, as suggested by Matsunaga et al. [9]. Interestingly, we observed a nonlinear relationship between cotinine and IL-1β, suggesting that IL-1β shows little to no response to low values of cotinine ranging up to 5.3 ng/mL. However, IL-1β values seem to increase potentially due to cotinine starting at 5.3 ng/mL and ranging up to 29.2 ng/mL. These findings need to be replicated to further examine how varying ETS exposure levels may influence the immune system.

We did not observe an association between ETS exposure levels and IL-8 or TNF-α or IL-10. In contrast to our findings, Wilson et al. [8] found lower levels of the pro-inflammatory cytokines IL-1β, IL-4, IL-5, and IFN-γ in healthy 1−6 year old children exposed to ETS compared to those with no ETS exposure, which suggests that ETS exposure had an immunosuppressive effect in that cohort. However, there were differences in Wilson and colleagues’ measures compared to our study as they assessed cytokines in blood samples, largely used a parental report of ETS exposure, and the children were illness-free. Further, in contrast of our finding that ETS exposure was associated with IL-1β, Matsunaga et al. [9] found no statistical differences with cotinine levels and IL-1β or IL-2, IL-4, IL-5, or IL-6 in healthy adolescents exposed to ETS. Their population differed from the current study as they were healthy, older (mean age 14.2 years), and had lower cotinine levels (GeoM = 0.82 [95% CI = −0.62−1.07]) compared to our sample (GeoM = 1.8 [95% CI = 1.4−2.2]). Additionally, cotinine and cytokines were measured in serum in this population; thus, their observed serum cytokine levels could be due to systemic inflammation present in these healthy children, and although cotinine levels in saliva are similar to that of serum, they are approximately 15%−40% higher [21,22]. Finally, we did not observe an association with IL-8 and ETS exposure, but Chahal et al. [7] found higher levels of the pro-inflammatory cytokine IL-8 in neonates with ETS exposure during pregnancy compared to unexposed neonates, and Riis et al. [10] found higher levels of TNF-α in five-year-olds with ETS exposure [10].

Although we did not find differences in ETS exposure and the mean levels of IL-8, TNF-α, and IL-10, we did find higher levels of certain cytokines in nonsmoking children with different discharge diagnoses in our multivariate regression model. While we assessed discharge diagnoses and potential interaction effects in our model, it is important to note that different diagnoses may be a potential confounder on the relationship between TSE and inflammatory markers in children. Thus, future research should consider using larger, more homogeneous samples of children with similar illness types to assess the potentially complex relationship between TSE and inflammatory markers so that the clinical relevance and application can be understood.

There are some limitations to be noted. First, we were not able to account for other factors that may have contributed to our results, including differences in the duration or sources of ETS exposure, differences in race or ethnicity, medication use, and the wide variety of illnesses in our population, which may have affected cytokine responses [4,5,6]. The timing of saliva collection may have affected both cotinine and cytokine levels [10,13]. However, Riis et al. [10] found that salivary cytokine levels were unrelated to sex, race, medication use, current illness, or recent health. Thus, Riis et al. indicated that salivary cytokine levels might reflect local immune processes of the oral cavity and not systemic inflammation, which increases the confidence of our results. Second, by using cross-sectional data, we did not account for differences that may have occurred with variations in ETS exposure over time. Third, we did not obtain information on concurrent medication use, and if the participants were on certain medications, such as corticosteroids, their immune response and subsequent cytokine response might have been inhibited [23]. Finally, because we included all hospitalized children and did not exclude those with asthma, infectious illnesses, or atopy, it is difficult to interpret the observed cytokine levels, which may have been the results of the illnesses themselves. To address this issue, we included all diagnoses in our models and examined the potential interactions of these diagnoses with cotinine. However, since the main objective was to examine the association of ETS exposure with inflammatory marker levels, we were able to achieve this goal with this unique population of ill children who had a wide range of ETS exposure.

## 5. Conclusions

In conclusion, we found that hospitalized, nonsmoking children are highly exposed to ETS and higher ETS exposure levels are associated with higher levels of the cytokine IL-1β, a pro-inflammatory cytokine linked to airway inflammation, regardless of child age, sex, and discharge diagnosis. To our knowledge, this is the first study showing this relationship in ill children. This suggests that ETS exposure increases pro-inflammatory immune responses in children, and may interfere with native immune responses and the body’s ability to heal and fight infection. This study also demonstrates that saliva can be used to measure cytokine levels in the saliva of hospitalized children; however, studies to compare these findings with cytokine levels in blood are necessary. Future prospective studies with larger sample sizes, standardized timing of collection, and evaluation of children with similar illnesses are necessary to compare with our findings so that different participant characteristics such as demographics, ETS exposure levels, and illness types can be evaluated and compared. Longitudinal research that assesses the relationships between ETS exposure and biomarkers of inflammation may provide unique insights into how TSE may augment or suppress specific inflammatory responses and potential associations with new diagnoses in children over time. Nevertheless, this study adds to research that emphasizes the need for strict ETS exposure control strategies to maintain children’s innate response to illness and to decrease preventable ETS exposure-related morbidity in children.

## Figures and Tables

**Table 1:** Patient characteristics by log-cotinine levels.

Characteristic	*n* (%)	Cotinine (ng/mL)		*p*–Value ^a^
		Geomean (95% CI)	Median (IQR)	
**Child Age Categories**	112 (100.0)	1.8 (1.4–2.2)	1.1 (0.6–2.9)	**0.004**
0–1 Years Old	41 (36.6)	2.6 (1.7–3.7)	1.9 (0.9–4.6)	Ref
2–4 Years Old	10 (8.9)	1.9 (0.8–3.8)	1.6 (0.9–2.9)	0.44
5–9 Years Old	28 (25.0)	**1.4 (0.8–2.1)**	**0.9 (0.5–2.0)**	**0.03**
10–17 Years Old	33 (29.5)	**1.2 (0.7–1.8)**	**0.9 (0.5–1.6)**	**0.006**
**Child Sex**				
Male	66 (58.9)	1.7 (1.2–2.2)	1.0 (0.6–2.4)	Ref
Female	46 (41.1)	1.9 (1.3–2.6)	1.4 (0.6–4.1)	0.61
**Child Race**				
White, non–Hispanic	87 (77.7)	1.9 (1.4–2.4)	1.1 (0.6–3.2)	Ref
African–American, non–Hispanic	17 (15.2)	1.3 (0.7–1.8)	1.0 (0.7–1.8)	0.19
Other	8 (7.1)	1.9 (0.8–3.7)	1.4 (0.9–2.6)	0.98
**Insurance Type**				
Public/Self–Pay	65 (58.0)	**2.7 (2.0–3.5)**	**2.0 (0.9–5.2)**	**<0.001**
Commercial	47 (42.0)	0.8 (0.6–1.1)	0.7 (0.4–1.0)	Ref
**Household Income Level**				
<$15,000	30 (27.0)	**3.5 (2.3–5.1)**	**3.0 (1.2–6.3)**	**<0.001**
>$15,000	81 (73.0)	1.3 (1.0–1.6)	0.9 (0.5–1.8)	Ref
**Discharge Diagnosis**				
Asthma or Asthma/Wheezing	24 (21.4)	2.0 (1.3–2.9)	1.7 (0.8–4.0)	Ref
Viral or Bronchiolitis/Wheezing	39 (34.8)	1.9 (1.3–2.7)	1.6 (0.7–3.0)	0.88
Bacterial Illness and/or Pneumonia	26 (23.2)	1.6 (0.8–2.6)	0.9 (0.5–1.7)	0.47
Non–Inflammatory Other	19 (17.0)	1.4 (0.7–2.4)	0.9 (0.5–1.6)	0.39
Inflammatory Other	4 (3.6)	1.7 (−0.7–24.9)	0.5 (0.4–2.0)	0.84

N = 112; missing values excluded; EHR, electronic health record; bold print indicates statistical significance *p* < 0.05. ^a^ *p*-values are unadjusted.

**Table 2:** Multivariate regression model results assessing the relationship between cotinine and inflammatory markers ^a^.

Inflammatory Markers and Diagnoses	$\hat{\beta }$	95% CI (Upper, Lower)	*p*-Value
**IL-1β**			
Cotinine (linear term)	**−1.12**	**−2.08, 0.15**	**0.024**
Cotinine (quadratic term)	**1.14**	**0.42, 1.86**	**0.002**
**Diagnosis ^b^**			
Asthma	0.19	−0.09, 0.47	0.17
Bronchiolitis	0.09	−0.17, 0.34	0.50
Bacterial/Pneumonia	0.14	−0.13, 0.41	0.30
Inflammatory Other	−0.49	−1.00, 0.01	0.06
**IL-10**			
Cotinine (linear term)	−0.30	−0.86, 0.25	0.28
Cotinine (quadratic term)	0.21	−0.20, 0.63	0.31
**Diagnosis**			
Asthma	−0.11	−0.27, 0.05	0.17
Bronchiolitis	−0.04	−0.18, 0.11	0.60
Bacterial/Pneumonia	−0.08	−0.24, 0.08	0.32
Inflammatory Other	**−0.45**	**−0.74, −0.16**	**0.003**
**IL-8**			
Cotinine (linear term)	−0.60	−1.51, 0.32	0.20
Cotinine (quadratic term)	0.48	−0.20, 1.16	0.16
**Diagnosis**			
Asthma	**0.34**	**0.08, 0.61**	**0.01**
Bronchiolitis	0.22	−0.02, 0.46	0.07
Bacterial/Pneumonia	**0.40**	**0.14, 0.66**	**0.002**
Inflammatory Other	−0.16	−0.64, 0.32	0.51
**TNF-α**			
Cotinine (linear term)	−0.30	−1.04, 0.43	0.42
Cotinine (quadratic term)	0.34	−0.20, 0.89	0.22
**Diagnosis**			
Asthma	0.13	−0.08, 0.34	0.22
Bronchiolitis	−0.07	−0.26, 0.12	0.46
Bacterial/Pneumonia	0.15	−0.06, 0.35	0.16
Inflammatory Other	**−0.52**	**−0.91, −0.13**	**0.009**

^a^ Inflammatory markers and cotinine were log-transformed for analyses; ^b^ reference category is non-inflammatory other; bold print indicates statistical significance *p* < 0.05.

**Table 3:** Correlations between log-cotinine and log-inflammatory marker values of hospitalized children.

Variables	Correlation Coefficient	*p*-Value
**Age (years)**	**−0.273**	**0.004**
**Inflammatory Markers ^a^**		
IL-1β (pg/mL)	**0.225**	**0.02**
IL-10 (pg/mL)	−0.029	0.76
IL-8 (pg/mL)	0.008	0.93
TNF-α (pg/mL)	0.130	0.17

N = 112; ^a^ Inflammatory markers were log-transformed for analyses; bold print indicates statistical significance *p* < 0.05.

**Table 4:** Inflammatory marker value differences between the lower 25th percentile and upper 75th percentiles of log-cotinine values ^a^.

Inflammatory Markers	Lower 25th Percentile of Salivary Cotinine Levels (0.075−0.59 ng/mL)		Upper 75th Percentile of Salivary Cotinine Levels (2.9−29.2 ng/mL)		*p*−Value
	*n*	Geomean (95% CI)	*n*	Geomean (95% CI)	
IL-1β (pg/mL)	28	254.0 (178.9−360.4)	28	282.2 (161.3−493.4)	0.74
IL-10 (pg/mL)	28	1.8 (1.1−2.7)	28	1.3 (0.8−1.9)	0.33
IL-8 (pg/mL)	28	829.16 (591.8−1161.6)	28	710.58 (464.6−1086.5)	0.56
TNF-α (pg/mL)	28	9.20 (6.3−13.3)	28	9.7 (6.6−14.2)	0.83

^a^ Inflammatory markers and cotinine were log-transformed for analyses.
